# *Rehmannia glutinosa* exhibits anti‐aging effect through maintaining the quiescence and decreasing the senescence of hematopoietic stem cells

**DOI:** 10.1002/ame2.12034

**Published:** 2018-10-24

**Authors:** Lin Bai, Gui‐ying Shi, Ya‐jun Yang, Wei Chen, Lian‐feng Zhang, Chuan Qin

**Affiliations:** ^1^ Key Laboratory of Human Disease Comparative Medicine Ministry of Health Institute of Laboratory Animal Science Chinese Academy of Medical Sciences and Comparative Medical Center Peking Union Medical College Beijing China

**Keywords:** anti‐aging, hematopoietic stem cells, quiescence, *Rehmannia glutinosa*

## Abstract

**Background:**

The time‐related decline in regenerative capacity and organ homeostasis is a major feature of aging. *Rehmannia glutinosa* and *Astragalus membranaceus* have been used as traditional Chinese herbal medicines for enhanced immunity and prolonged life. However, the mechanism by which this herbal medicine slows aging is unknown. In this study, we investigated the mechanism of the herbal anti‐aging effect.

**Methods:**

Mice were fed diets supplemented with *R. glutinosa* or *A. membranaceus* for 10 months; the control group was fed a standard diet. The phenotypes were evaluated using a grading score system and survival analysis. The percentages of the senescence phenotypes of hematopoietic stem cells (HSCs) were determined by fluorescence‐activated cell sorting analysis. The function and the mechanism of HSCs were analyzed by clonogenic assay and the real‐time polymerase chain reaction.

**Results:**

The anti‐aging effect of *R. glutinosa* is due to the enhanced function of HSCs. Mice fed with *R. glutinosa* displayed characteristics of a slowed aging process, including decreased senescence and increased rate of survival. Flow cytometry analysis showed decreased numbers of Lin^–^Sca1^+^c‐kit^–^ (LSK) cells, long‐term HSCs (LT‐HSCs) and short‐term HSCs (ST‐HSCs) in the *R. glutinosa* group. In vitro, clonogenic assays showed increased self‐renewal ability of LT‐HSCs from the *R. glutinosa* group as well as maintaining LSK quiescence through upregulated p18 expression. The *R. glutinosa* group also showed decreased reactive oxygen species levels and the percentage of β‐gal^+^ cells through downregulation of the cellular senescence‐associated protein p53 and p16.

**Conclusion:**

*Rehmannia glutinosa* exerts anti‐aging effects by maintaining the quiescence and decreasing the senescence of HSCs.

## 1 INTRODUCTION

Aging is defined as the time‐dependent functional decline that affects most living organisms.^1^ The longevity of organisms is mainly maintained by stem cells.^2^ Tissue‐specific stem cells have the capacity for self‐renewal and differentiate into a variety of effector cells.^3^ However, during the aging process, this self‐renewal capacity declines, eventually leading to the accumulation of unrepaired, damaged tissues in aged organisms.^4^ Loss of the ability to maintain a balance between quiescence and differentiation in the stem/progenitor cell compartment may result in death or age‐associated diseases.^2^ Furthermore, recent studies suggest that stem cell rejuvenation may reverse the aging phenotype.^5^, ^6^ In the hematopoietic system, hematopoietic stem cells (HSCs) are responsible for blood cell production. In aged mice, the hematopoietic system shows T‐ and B‐lymphoid cell impairment and the number of myeloid cells is increased. Aged HSCs showed reduced self‐renewal activity and reduced hematopoiesis reconstructive ability. Notably, age‐related changes in the HSC compartment are manifested in the aging host as anemia, increased propensity for myeloproliferative neoplasms, decreased immune function and increased cancer incidence.^7^, ^8^, ^9^ However, the mechanism of HSC aging and the effects of herbal medicines on HSC aging are unknown.^10^

People have been concerned with delaying the aging process and staying young from ancient times and anti‐aging is a current focus of research. Traditional Chinese medicine has received increasing attention for the treatment of various aging‐associated diseases. Many herbs used in traditional Chinese medicine are known to provide positive effects against aging through different mechanisms. *Rehmannia glutinosa* and *Astragalus membranaceus* have been widely used in this way for thousands of years.

*Rehmannia glutinosa* is used to treat various diabetic disorders, enhance the bone metabolism in osteoporosis, and inhibit liver inflammation and fibrosis. In addition, this herb has other effects including anti‐fatigue, antidepressant and neuroprotective properties. In the past few years, pharmacological studies on *R. glutinosa* and its active components have focused mainly on its broad actions on the blood, endocrine, cardiovascular and nervous systems.^11^, ^12^, ^13^, ^14^ Thus, *R. glutinosa* was shown to possess strong immuno‐enhancement activity, which has provided the theoretical basis for further studies.

*Astragalus membranaceus* possesses tonic, hepatoprotective, diuretic and expectorant properties^15^ and has been shown to exhibit immunomodulatory,^16^ anti‐inflammatory and antioxidant effects.^17^ The elucidation of the molecular mechanisms underlying the effects of traditional Chinese medicines in clinical practice is a key step toward their worldwide application, and this topic is currently a subject of intense research interest.

Thus, in this study, we fed mice diets supplemented with *R. glutinosa* and *A. membranaceus* for 10 months to explore the mechanism underlying the ability of *R. glutinosa* to increase longevity.

## 2 MATERIALS AND METHODS

### 2.1 Animal grouping and treatment

C57BL/6J female mice were maintained in a pathogen‐free environment and fed with a standard diet. The use of animals in this study was approved by the Animal Care and Use Committees of the Institute of Laboratory Animal Science of Peking Union Medical College. The mice (aged 10 months) were randomly divided into three groups (n = 20/group). The control group was fed with a standard diet (Beijing HFK Biosicence, Beijing, China). The diets of the other two groups were supplemented with ground *R. glutinosa* and *A. membranaceus* (Beijing Tong Ren Tang Chinese Medicine, Beijing, China), respectively, at a dose of 200 mg/d for 10 months. This dose was selected using the body surface area normalization method to confirm the drug doses from human studies to mouse studies. The bodyweight was determined every 2 months and survival was recorded daily.

### 2.2 Evaluation of the degree of senescence

A grading score system was adopted to evaluate the degree of senescence according to criteria defined by Takeda et al.^18^ Each category listed in the protocol was selected from the clinical signs associated with the aging process. Each mouse was scored at 18 months of age and the score in each category was summed to determine the overall grading score.

### 2.3 Flow cytometry

Cells were harvested from the thymus, spleen, peripheral blood (PB) and bone marrow (BM). The spleen and thymus were excised immediately, washed with saline and weighed. Spleens and thymuses were gently homogenized in a glass homogenizer and cells were suspended in sterile phosphate‐buffered saline (PBS). The cells from PB were applied to blood red cell lysis (BD Biosciences, San Jose, CA, USA). The cells from BM were isolated by flushing both tibias and femurs with sterile PBS. All the cells were isolated by filtration across a sterile nylon mesh and stained for 30 minutes at 4°C with the following fluorophore‐conjugated antibodies: phycoerythrin (PE)‐conjugated anti‐CD3 (G4.18), allophycocyanin (APC)‐conjugated anti‐CD4 (OX35) and PE‐Cy7‐conjugated anti‐CD8a (OX8). For the BM cells, lineage markers were stained using biotin‐conjugated antimouse CD4 (RM4‐5), CD5 (53‐7.3) CD8a (53‐6.7), CD11b (M1/70), B220 (RA3‐6B2), TER119 (TER‐119) and Gr1 (RB6‐8C5), followed by staining with the antibody APC‐eFluor 780‐conjugated streptavidin was also obtained from eBioscience (San Diego, CA, USA). The following antibodies were used for surface staining: APC immunoglobulin (Ig)M (II/41), fluorescein isothiocyanate (FITC) IgD (11‐26), PE‐Cy7 Sca‐1 (D7), PE Flt3 (A2F10), FITC B220 (RA3‐6B2), FITC CD34 (RAM34), PerCP‐Cy5.5 CD127 (A7R34), PE CD16/CD32 (93) and PerCP‐Cy5.5 CD3e (145‐2C11). All antibodies were obtained from eBiosciences (San Diego, CA, USA). Data were acquired by a FACS Aria II (Becton Dickinson, Franklin Lakes, NJ, USA) and analyzed using FlowJo software (Three Star, Ashland, OR, USA).

### 2.4 Cell cycle analysis

For cell cycle analysis, total BM cells were stained for stem cell surface markers (Lin^–^Sca‐1^+^c‐Kit^High^; LSTKs), then fixed and permeabilized (00‐5123‐43; Becton Dickinson) before staining with FITC Ki‐67 antibody and 7‐aminoactinomycin D (7‐AAD). Data acquisition was performed on FACS Aria II (Becton Dickinson). Data were analyzed using FlowJo software.

### 2.5 Senescence‐associated β‐gal staining

Senescence‐associated (SA)‐β‐gal activity was also assayed by flow cytometry using C_12_FDG as described by the manufacturer (Molecular Probes, Eugene, OR, USA). Briefly, stem cells were first stained for cell surface markers and then incubated with 100 nmol L^−1^ bafilomycin A1 for 1 hour at 37°C to induce lysosomal alkalinization. After washing with PBS, cells were incubated with a 2 mmol L^−1^ C_12_FDG for 1‐2 hours at 37°C and then analyzed using a FACS Aria I (Becton Dickinson).

### 2.6 Determination of reactive oxygen species (ROS) production

Cells were incubated with dichloro‐dihydro‐fluorescein diacetate (DCFH‐DA; Beyotime, Shanghai, China) at 37°C for 20 minutes. DCFH‐DA diffuses passively into cells, where it is deacetylated by esterases to form non‐fluorescent 2′,7′‐dichlorofluorescein (DCFH). The amount of fluorescence emitted correlates with the quantity of ROS in the cell. Data were acquired on a FACS Aria I (Becton Dickinson) and analyzed using FlowJo software.

### 2.7 Real‐time polymerase chain reaction (PCR) analysis

Total RNA was extracted from cells using TRIzol reagent (Invitrogen, San Diego, CA, USA) according to the manufacturer's instructions. Genes of interest were amplified from DNase I‐treated total RNAs using M‐MLV Reverse Transcriptase (Promega, Madison, WI, USA) and poly‐dT primers. The primers used for PCR were as follows: p21 (5′‐TCCAGACATTCAGAGCCACA‐3′ and 5′‐CGAAGAGACAACGGCACACT‐3′, Tm = 60°C, 30 cycles), p53 (5′‐CATGAACCGCCGACCTATC‐3′ and 5′‐TCCCGGAACATCTCGAGGC‐3′, Tm = 62°C, 35 cycles), p16 (5′‐CGAACTCTTTCGGTCGTACCC‐3′ and 5′‐CGAATCTGCACCGTAGTTGAGC‐3′, Tm = 62°C, 35 cycles), p57 (5′‐AGGAGCAGGACGAGAATCAA‐3′ and 5′‐ TTCTCCTGCGCAGTTCTC TT‐3′, Tm = 61°C, 30 cycles), p19 (5′‐ATGGGTCGCAGGTTCTTGGT‐3′ and 5′‐GTAGTGGGGTCCTCGCAGTT‐3′, Tm = 61°C, 35 cycles), p18 (5′‐GGGACCTAGAGCAACTTACT‐3′ and 5′‐TGACAGCAAAACCAGTTCCA‐3′, Tm = 61°C, 30 cycles), glyceraldehyde 3‐phosphate dehydrogenase (5′‐GAGCGAGACCCCACTAACAT‐3′ and 5′‐TTCACACCCATCACAAACAT‐3′, Tm = 60°C, 25 cycles). Real‐time (RT)‐PCR was carried using SYBR Premix Ex Taq II (TaKaRa Shuzo, Kyoto, Japan) on the ABI StepOne™ detection system (Applied Biosystems, Foster City, CA, USA).

### 2.8 Clonogenic assays

Long‐term (LT)‐HSCs were sorted by fluorescence‐activated cell sorting (FACS) and then cultured in 96‐well cell plates using a methylcellulose‐base medium (HSC007; R&D Systems, Minneapolis, MN, USA). Ten replicate wells were prepared for each sample. We used 96‐well cell culture plate, three cells per well. Two weeks after plating, the number and sizes of colonies were counted under a microscope.

### 2.9 Statistical analysis

Data were analyzed by the one‐way anova using Microsoft Excel (Microsoft, Redmond, WA, USA) and GraphPad Prism (GraphPad Software, La Jolla, CA, USA) software. Data were presented as the mean ± SD. *P *< 0.05 was considered to indicate statistical significance.

## 3 RESULTS

### 3.1 *R. glutinosa* and *A. membranaceus* exerted anti‐aging effects

**Figure 1:**
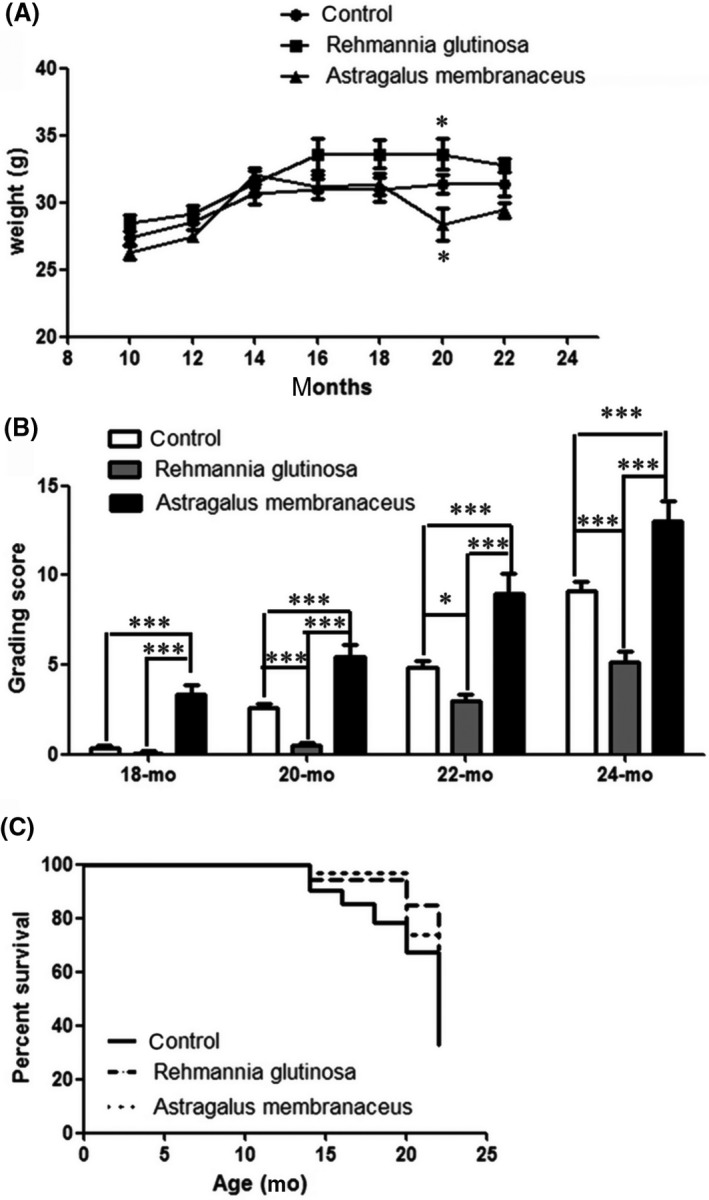
*Rehmannia glutinosa* and *Astragalus membranaceus* had anti‐aging effects. Mice were fed diets supplemented with ground *R. glutinosa* or *A. membranaceus* (200 mg/d); the control group was fed a standard diet. A, Changes in bodyweight of mice measured every 2 months. B, Changes in the senescence grading score of mice with age. C, Survival curves. Data represent the mean ± SD; n = 20 mice/group. Data represent the mean ± SD; n = 5 mice/group. *P < 0.05, ***P < 0.001

To confirm the anti‐aging effects of *R. glutinosa* and *A. membranaceus*, we investigated the effects of administration as a dietary supplement (200 mg/d). Subsequently, we recorded the bodyweight, degree of senescence and survival rate of mice. The bodyweight of mice feeding with *R. glutinosa* or *A. membranaceus* was normal compared with those in the control group, although there was a decrease in the bodyweight of the *R. glutinosa* and *A. membranaceus* group at 20 months (Figure 1A). Analysis of the degree of senescence revealed a steady and irreversible increase in the grading score with advancing age in the *R. glutinosa* and *A. membranaceus* groups compared with that in the control group, although the increase was more marked in the *A. membranaceus* group (Figure 1B). The high grading score was due to an earlier onset of loss of passivity and reactivity, loss of skin glossiness and increased coarseness, hair loss, periophthalmic lesions, increased lordokyphosis of the spine and a more marked increase in their severity.^18^ Mice in the *A. membranaceus* mice group showed more marked senescence phenotype characteristics, including loss of skin glossiness and hair loss. The survival curves showed that the lifespan of mice in the *R. glutinosa* and *A. membranaceus* groups was slightly longer than that in the control group (Figure 1C). These data confirmed the anti‐aging effects of both *R. glutinosa* and *A. membranaceus*, although *R. glutinosa* was found to be more efficient in slowing the aging process.

### 3.2 *R. glutinosa* reduced the number of hematopoietic stem cells

**Figure 2:**
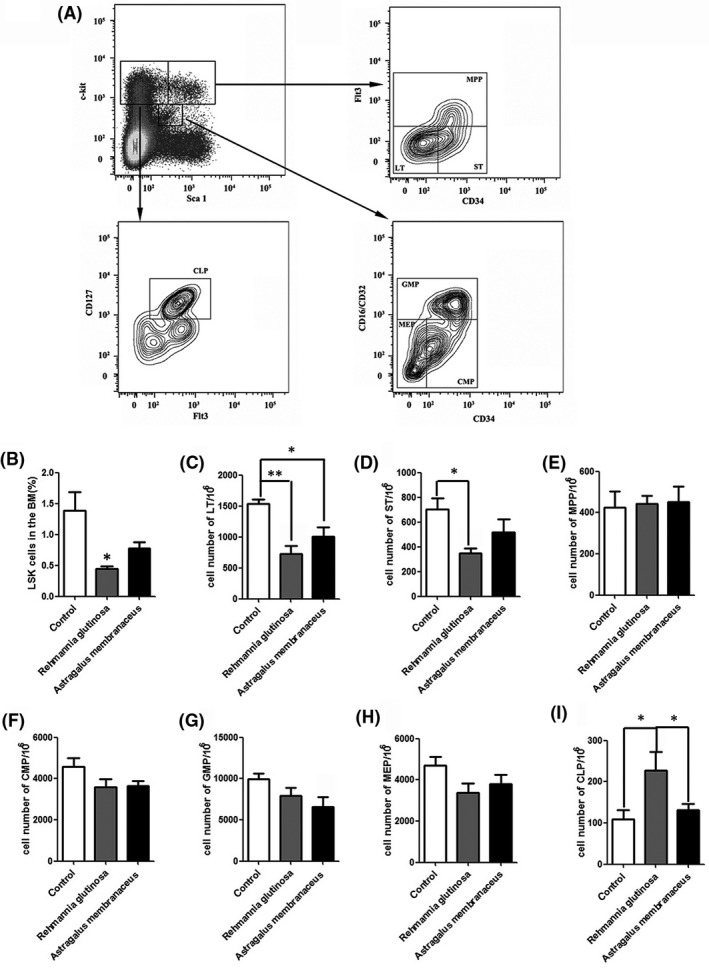
*Rehmannia glutinosa* and *Astragalus membranaceus* affected the hematopoietic stem/progenitor cells number. Mice (aged 20 months) were fed diets supplemented with *R. glutinosa* or *A. membranaceus* (200 mg/d) for 10 months (n = 5/group); the control group was fed a standard diet. Freshly isolated bone marrow (BM) cells were stained with the indicated antibodies and analyzed by flow cytometry. A, Representative staining profiles of BM hematopoietic stem cell and progenitor cell populations. B, The percentage of Lin^–^Sca1^+^c‐kit^–^ cells (LSKs) in BM cells. C‐I, Cell numbers of (C) long‐term (LT; Lin^–^, Sca‐1^+^, c‐Kit^+^, CD34^–^, Flt3^–^); D, short‐term (ST; Lin^–^, Sca‐1^+^, c‐Kit^+^, CD34^+^, Flt3^–^); E, multipotent progenitor (MPP; Lin^–^, Sca‐1^+^, c‐Kit^+^, Flt3^+^); F, common myeloid progenitor (CMP; Lin^–^, Sca‐1^–^, c‐Kit^–^, CD34^+^, CD16/CD32^–^); G, granulocyte‐macrophage progenitor (GMP; Lin^–^, Sca‐1^–^, c‐Kit^–^, CD34^+^, CD16/CD32^+^); H, megakaryocyte‐erythroid progenitor (MEP; Lin^–^, Sca‐1^–^, c‐Kit^–^, CD34^–^, CD16/CD32^–^); and I, common lymphoid progenitor (CLP; Lin^–^, Sca‐1^low^, c‐Kit^low^, CD127^+^). Data represent the mean ± SD; n = 5 mice/group. *P < 0.05, **P < 0.01

Stem cell exhaustion is thought to be the integrative consequence of multiple types of aging‐associated damage and one of the major causes of tissue and organism aging.^1^ Recent studies suggest that stem cell rejuvenation may reverse the aging phenotype at the organism level.^5^ We hypothesized that the anti‐aging effects of *R. glutinosa* are mediated by enhancing stem cell function. Therefore, we performed flow cytometric analysis of the number of hematopoietic stem/progenitor cells isolated from mice (aged 20 months) fed diets supplemented with *R. glutinosa* or *A. membranaceus* (200 mg/d) for 10 months (Figure 2A). The percentage of LSKs was reduced in the *R. glutinosa* and *A. membranaceus* groups (Figure 2B). The numbers of LT‐ and short‐term (ST)‐HSCs were reduced by approximately twofold in the *R. glutinosa* group compared with the number in the control group (Figure 2C‐D). Greater numbers of common lymphoid progenitors (CLPs) were detected in the *R. glutinosa* group compared with the number in the control group (Figure 2I). There were no differences in the numbers of multipotent progenitors (MPPs), common myeloid progenitors (CMPs), granulocyte‐macrophage progenitors (GMPs) and megakaryocyte‐erythroid progenitors (MEPs) in the *R. glutinosa* group compared with the number in the control group. In the *A. membranaceus* group, there was no significant difference in the numbers of LT‐ and ST‐HSCs compared with the numbers in the control group (Figure 2C‐D). Furthermore, there was no difference in the numbers of MPP, CMP, MEP and CLP cells compared with the numbers in the control group (Figure 2E, 2F, 2H and 2I); however, the number of GMPs was decreased in the *A. membranaceus* group. Thus, mice fed diets supplemented with *R. glutinosa* exhibited decreased numbers of hematopoietic stem and progenitor cells.

### 3.3 *R. glutinosa* enhanced the function and maintained the quiescence of HSCs

**Figure 3:**
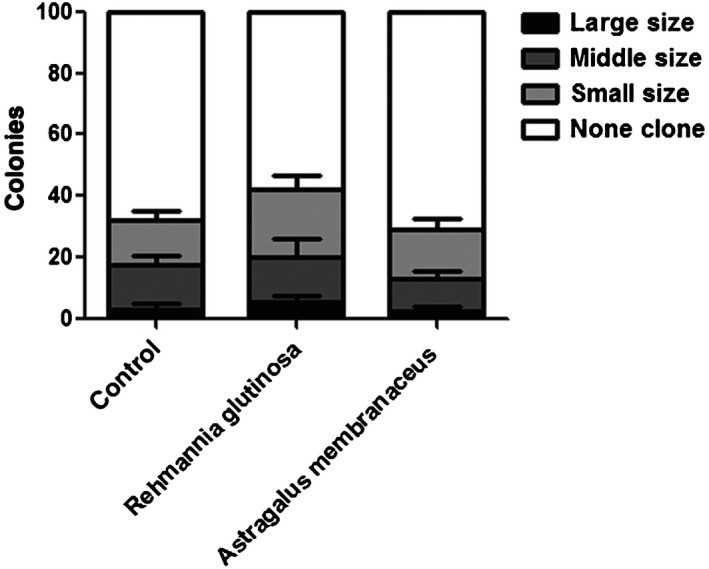
Function of hematopoietic stem cells enhanced by dietary *Rehmannia glutinosa* supplementation. In vitro clonogenic potential of long‐term hepatic stellate cells from mice (n = 3)

To evaluate the function of HSCs, the clonogenic potential of LT‐HSCs was examined in vitro. We sorted LT‐HSCs cells into a methylcellulose‐base medium using FACS and counted the number and size of colonies after 14 days. Compared with the number and size of colonies produced by cells from mice in the control group, cells from mice in the *R. glutinosa* group displayed an increase in the number of colonies, especially for the small size (*P = *0.0241) and large size clone (*P = *0.0418), while there was no difference in the number from mice in the *A. membranaceus* group (Figure 3). The increased colony size and number in the *R. glutinosa* group suggested that this herb enhances the self‐renewal potential of LT‐HSCs.

**Figure 4:**
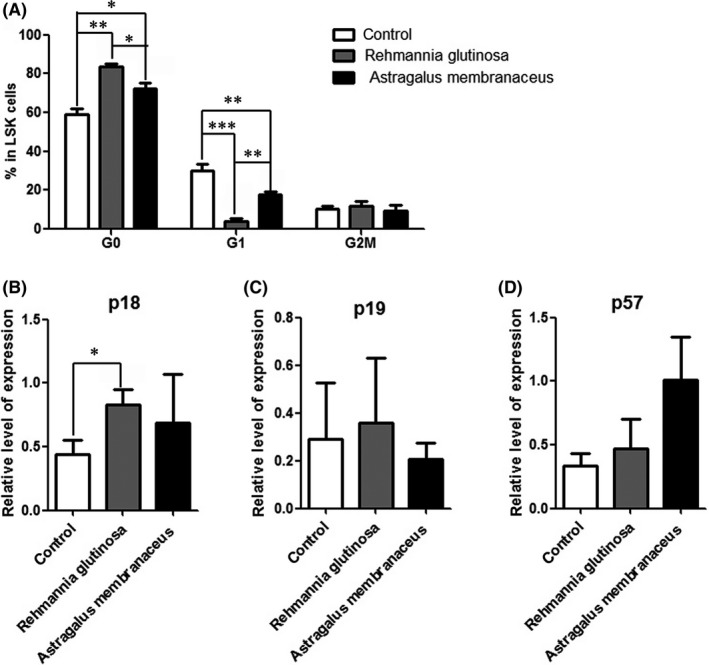
Dietary supplements of *Rehmannia glutinosa* and *Astragalus membranaceus* maintained the quiescence of hematopoietic stem cells. A, The percentage of cells in each phase of the cell cycle. Flow cytometric analysis of 7‐aminoactinomycin D (7‐AAD) and Ki‐67 staining of Lin^–^Sca1^+^c‐kit^–^ cells (LSKs). B, Real‐time polymerase chain reaction analysis of sorted LSK cell gene expression; *GAPDH* was used for normalization. Data represent the mean ± SD of three experiments. *P < 0.05, **P < 0.01, ***P < 0.001

Most HSCs in adult mice remain quiescent;^19^ therefore, the maintenance of cellular quiescence is an essential mechanism for stem cell self‐renewal.^20^, ^21^ The observed decreases in HSCs suggest diminished cell proliferation in the *R. glutinosa* and *A. membranaceus* groups; therefore, we examined the cell cycle status of HSCs through analysis of the proliferative cell marker Ki‐67 combined with determination of the DNA content 7‐AAD in the LSK population. We observed an increased number of LSK cells in the G0 phase in the *R. glutinosa* and *A. membranaceus* groups compared with that in the control group (Figure 4A). These results indicated that *R. glutinosa* and *A. membranaceus* maintained the quiescence of HSCs.

Real‐time PCR analysis of cell cycle regulators in LSK cells revealed that p18, p19 and p57 played critical roles in the maintenance of HSC quiescence.^22^, ^23^, ^24^ There were no obvious changes observed for p57 and p19 (Figure 4C‐D), and p18 was upregulated in the *R. glutinosa* group compared with that in the control group, and slightly upregulated in the *A. membranaceus* group (Figure 4B).

### 3.4 *R. glutinosa* delayed HSCs senescence

**Figure 5:**
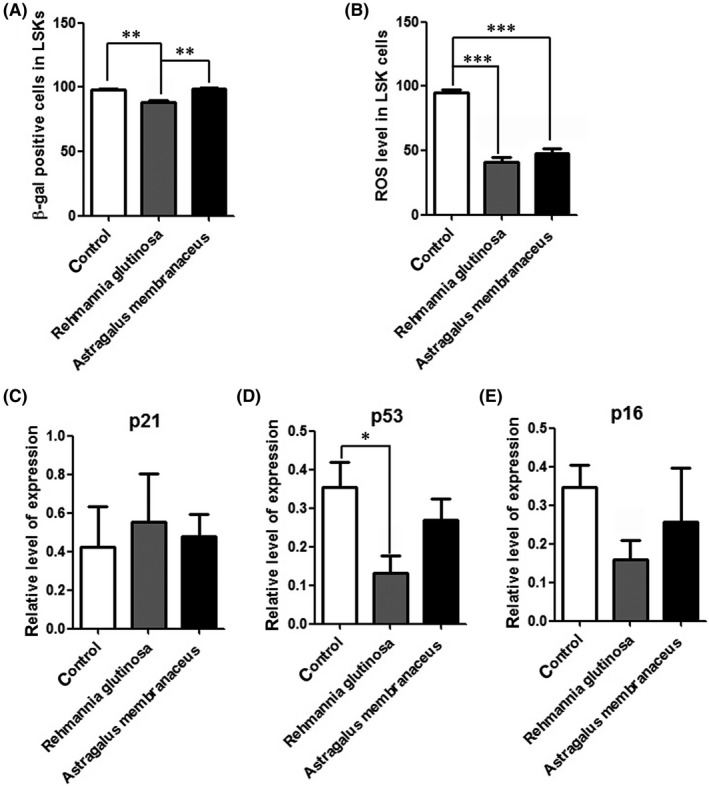
Dietary *Rehmannia glutinosa* supplementation reduced the hepatic stellate cell (HSC) senescence. Mice (aged 20 months) were fed diets supplemented with *Rehmannia glutinosa* or *Astragalus membranaceus* (200 mg/d) for 10 months (n = 5/group); the control group was fed a standard diet. A, Flow cytometric analysis of SA‐β‐gal‐positive cells Lin^–^Sca1^+^c‐kit^–^ (LSK) using a fluorescent β‐galactosidase substrate (C_12_ FDG). B, The percentage of reactive oxygen species (ROS)‐positive cells in LSKs. Flow cytometric analysis of ROS‐positive LSKs. Data represent the mean ± standard deviation (SD). *P < 0.05, ***P < 0.001. C, The expression pattern of cellular senescence‐associated genes in LSKs; *GAPDH* was used for normalization. Data represent the mean ± SD of three experiments. *P < 0.05, **P < 0.01, ***P < 0.001

Long‐term dietary supplementation with *R. glutinosa* can extend the lifespan of mice. Cellular senescence increases with age; therefore, we investigated HSC senescence in the aging mice by flow cytometric analysis of SA‐β‐gal stained HSCs using a fluorescent β‐gal substrate (C_12_FDG).^25^ The percentage of SA‐β‐gal‐positive cells was decreased in the *R. glutinosa* group, indicating that the *R. glutinosa* delays LSK cell senescence (Figure 5A).

Reactive oxygen species play a major role in HSC senescence, and loss of HSC quiescence is frequently correlated with increased cellular ROS.^26^ Analysis of the intracellular ROS in LSKs showed that the levels were decreased in the *R. glutinosa* group compared with those in the control group (Figure 5B). These data indicated that *R. glutinosa* may maintain HSC quiescence and enhance HSC function by decreasing ROS levels.

We then investigated the expression several genes involved in cellular senescence by RT‐PCR analysis of LSK cells. Compared with the control group, the expression of cellular senescence‐associated proteins p53 and p16 was downregulated in the *R. glutinosa* group (Figure 5D‐E). But the expression of p53 and p16 was not significantly downregulated between the *A. membranaceus* group and control group (Figure 5D‐E). Taken together, these data show that several key cell cycle and cell senescence‐related genes regulate the function of HSCs in mice fed diets supplemented with *R. glutinosa*.

### 3.5 *R. glutinosa* enhanced B‐cell immunity

B and T lymphocytes are both important for immunological responses. T lymphocytes play a critical role in the cellular immunity and its regulation, while B lymphocytes participate mainly in humoral immunity. Lymphocyte proliferation is the most immediate index reflecting organic immunity. To investigate the role in immunological enhancement, the effects of *R. glutinosa* and *A. membranaceus* on lymphocyte proliferation were examined.

**Figure 6:**
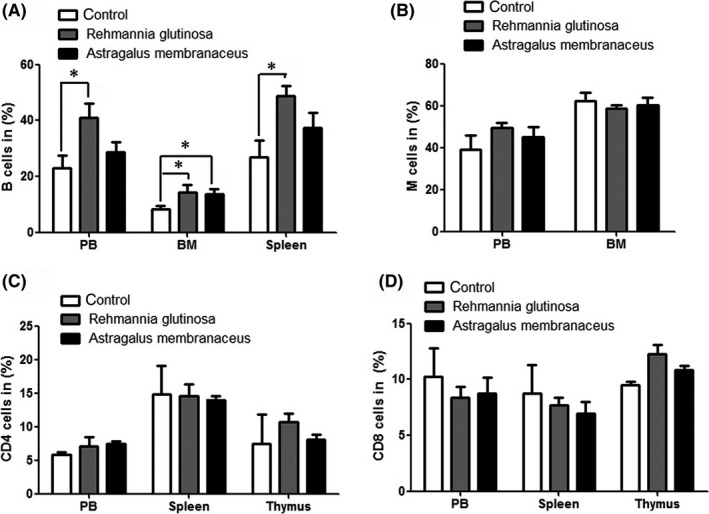
Flow cytometric analysis of the percentage of mature immune cells in peripheral blood (PB), bone marrow (BM), spleen and thymus. Mice (aged 20 months) were fed diets supplemented with *Rehmannia glutinosa* or *Astragalus membranaceus* (200 mg/d) for 10 months (n = 5/group); the control group was fed a standard diet. A, Flow cytometric analysis of the percentage of B cells (B220^+^) in PB, BM and spleen. B, Flow cytometric analysis of the percentage of monocyte and granulocyte cells (CD11b^+^) in PB and BM. C and D, Flow cytometric analysis of the percentage of CD4^+^ and CD8^+^ cells in PB, spleen and thymus. Data represent the mean ± SD. *P < 0.05

Mice in the *R. glutinosa* group exhibited decreased numbers of HSCs and increased numbers of CLPs. Flow cytometric analysis showed increased numbers of mature B cells (B220^+^) in the PB, BM and spleen of mice in the *R. glutinosa* group compared with the numbers detected in the control group (Figure 6A); however, there were no significant differences in the numbers of T cells (CD4^+^ and CD8^+^), monocytes and granulocytes (CD11b^+^) between the two groups (Figure 6B‐D). Thus, these results indicate that dietary *R. glutinosa* enhances B‐cell immunity.

## 4 DISCUSSION

In this study, we investigated the mechanism of the anti‐aging effects of *R. glutinosa* or *A. membranaceus* by supplementing the diets of mice with the herbs at a dose of 200 mg/d for 10 months. *R. glutinosa* was found to exhibit anti‐aging effects, including decreased senescence and increased survival of HSCs as well as enhanced B‐cell immunity. In terms of the mechanism of the anti‐aging effects, we found that the *R. glutinosa* decreased the number of HSCs, while the proliferation capacity was increased. Furthermore, *R. glutinosa* maintained HSC quiescence and decreased the numbers of SA‐β‐gal‐positive cells and ROS levels through regulation of p18, p53 and p16. In combination, our results confirmed that the anti‐aging effects of *R. glutinosa* in mice are medicated by maintaining the quiescence and enhancing the function of HSCs.

*Rehmannia glutinosa*, which has been used as traditional Chinese herbal medicine for thousands of years, can be used to treat hypoglycemia in various diabetic disorders.^27^, ^28^ It has also been reported that *R. glutinosa* extract enhances bone metabolism.^29^ Furthermore, *R. glutinosa* inhibits inflammatory responses and syndromes,^30^, ^31^ and protects against cell damage by scavenging free radicals.^14^, ^32^ In this study, we found that mice dietary supplementation with *R. glutinosa* exerted anti‐aging effects and enhanced B‐cell immunity by increasing the proliferative capacity of LT‐HSCs and decreasing the numbers of SA‐β‐gal‐positive cells and ROS levels.

The degree of oxidative damage has been found to increase with age in a variety of cells and tissues. Oxidative stress is a critical determinant of HSC self‐renewal. Loss of LT‐HSC quiescence frequently correlates with increased cellular ROS, which is negatively associated with HSCs self‐renewal.^26^ Catalpol is an iridoid glucoside, which has been found in the root of *R. glutinosa*. Catalpol showed inhibiting oxidative stress, DNA damage and telomere shortening through PGC‐1α/TERT pathway in a previous study.^33^ In our study, the level of ROS was decreased in the LSK cells from the *R. glutinosa* group compared with the control group. Thus *R. glutinosa* prolongs the survival of mice by inhibiting oxidative stress in the HSCs.

*Astragalus membranaceus* is also used in traditional Chinese medicine to promote immunity, reduce blood sugar and promote tumor cell apoptosis, and also has antioxidation and anti‐aging properties. In this study, we found that dietary supplementation with *A. membranaceus* for 10 months had no obvious effects compared with those observed in the control mice, while the weight of mice was reduced at 20 months. Therefore, *A. membranaceus* may be unsuitable for LT treatment to fed mice.

In conclusion, our results show that *R. glutinosa* can prolong the lifespan of mice by maintaining the quiescence and enhancing the function of HSCs. Furthermore, *R. glutinosa* can decrease the intercellular levels of ROS and the number of SA‐β‐gal‐positive cells and enhance B‐cell immunity.

## CONFLICTS OF INTEREST

None.

## AUTHOR CONTRIBUTIONS

All listed authors meet the requirements for authorship. CQ and LFZ conceived and designed the experiments. LB performed the experiments and wrote the main manuscript test. GYS performed and analyzed the data of FACS. YJY designed the experiment about the Chinese traditional Medicine. WC managed the mice. All authors have read and approved the manuscript.
